# Dr. Frederick A. Castle

**Published:** 1902-06

**Authors:** 


Dr. Frederick A. Castle, of New York, died April 28, 1902, aged 59 years. He served in the medical corps of the navy during the civil war and was a medical editor of prominence for many years. He also held the chair of materia medica and therapeutics in Bellevue Hospital Medical College for a considerable period. He was prominent in medical affairs generally, was a member of several clubs and a companion of the Military Order of the Loyal Legion.

